# Does the Number of Fingers on the Bag Influence Volume Delivery? A Randomized Model Study of Bag-Valve-Mask Ventilation in Infants

**DOI:** 10.3390/children5100132

**Published:** 2018-09-21

**Authors:** David Zweiker, Hanna Schwaberger, Berndt Urlesberger, Lukas P Mileder, Nariae Baik-Schneditz, Gerhard Pichler, Georg M Schmölzer, Bernhard Schwaberger

**Affiliations:** 1 Division of Neonatology, Department of Pediatrics and Adolescent Medicine, Medical University of Graz, Graz 8010, Austria; david.zweiker@medunigraz.at (D.Z.); hanna.kilbertus@gmail.com (H.S.); berndt.urlesberger@medunigraz.at (B.U.); lukas.mileder@medunigraz.at (L.P.M.); nariae.baik@medunigraz.at (N.B.-S.); gerhard.pichler@medunigraz.at (G.P.); georg.schmoelzer@me.com (G.M.S.); 2 Medizinercorps Graz, Austrian Red Cross Federal Association Styria, Graz 8010, Austria; 3 Centre for the Studies of Asphyxia and Resuscitation, Neonatal Research Unit, Royal Alexandra Hospital, Edmonton, AB T5H 3V9, Canada; 4 Department of Pediatrics, University of Alberta, Edmonton, AB T6G 1C9, Canada

**Keywords:** paediatric resuscitation, ventilation, non-invasive, mask ventilation, bag-valve-mask ventilation, ventilation technique

## Abstract

We sought to compare the effectiveness of two versus five fingers used for bag-valve-mask (BVM) ventilation on effective tidal volume (V_Teff_) delivery in an infant resuscitation model. In a randomised cross-over study, 40 healthcare professionals ventilated a modified leak-free infant resuscitation manikin with both two and five fingers, using a self-inflating bag. The delivered and effective tidal volumes, ventilation rate, and mask leak were measured and recorded using a respiratory function monitor. We found no significant differences in the V_Teff_ (five-finger 61.7 ± 23.9 vs. two-finger 58.8 ± 16.6 mL; *p* = 0.35) or ventilatory minute volume (2.71 ± 1.59 vs. 2.76 ± 1.24 L/min; *p* = 0.40) of both BVM ventilation techniques. However, there was an increase in the delivered tidal volume (V_Tdel_) and mask leak when using the five-finger technique compared with the two-finger technique (V_Tdel_ 96.1 ± 19.4 vs. 87.7 ± 15.5 mL; *p* < 0.01; and mask leak 34.6 ± 23.0 vs. 30.0 ± 21.0%; *p* = 0.02). Although the five-finger technique was associated with an increased mask leak, the number of fingers used during the BVM ventilation had no effect on V_Teff_ in an infant resuscitation model.

## 1. Introduction

Respiratory emergencies leading to hypoxaemia are the most frequent cause of pediatric cardiac arrest [1,2]. Furthermore, approximately 10% of newborn infants require respiratory support at birth [3,4]. Thus, the cornerstone of successful resuscitation in the pediatric population is adequate ventilation [5,6]. Bag-valve-mask ventilation (BVM) with self-inflating bags (SIBs) is the recommended first line of therapy for airway control and ventilation in children [5]. SIBs are widely used for pediatric and neonatal resuscitation, especially outside of the hospital or in resource-limited settings [7,8].

Healthcare personnel commonly provide excessive ventilation during cardio-pulmonary resuscitation [9,10], and this may be harmful [5]. Therefore, the correct BVM ventilation technique is essential in order to avoid hypo- and hyper-ventilation. For guidance, the current resuscitation guidelines mention adequate chest rise, mask holding techniques, and a correct head position during mask ventilation for newborn infants [5,6]. However, an optimal technique for holding the SIB is not provided. In pediatric emergency care courses, questions regarding the number of fingers that are to be used on the SIB for the delivery of an adequate tidal volume (V_T_) are often raised. Currently, there is only anecdotal evidence about the most effective holding technique when using SIBs. Current neonatal and pediatric emergency literature describes a “rule of thumb”, recommending the use of the thumb and one additional finger per kilogram body weight when using a 250 mL SIB [11,12,13]. Furthermore, a popular German midwifery book recommends the use of the thumb, index finger, and middle finger during BVM ventilation [14]. This diversity of recommendations leads to the uncertainty of healthcare personnel in training about the optimal number of fingers needed to hold a SIB. We aimed to examine and compare two different SIB holding techniques during a simulated resuscitation on an infant model. We hypothesized that the number of fingers used during BVM ventilation had an effect on the V_T_ delivery. Additionally, we were interested in the influence of the participants’ anthropometric parameters on the V_T_ delivery.

## 2. Materials and Methods

### 2.1. Participants

A randomized controlled pilot cross-over study was performed using 40 healthcare professionals. The Regional Committee on Biomedical Research Ethics of the Medical University of Graz (26-582 ex 13/14) approved the study. Pediatric intensive care nurses from the University Hospital at the Medical University of Graz, Austria, and medical students volunteering as paramedics were invited to participate in the study. The registered nurses were all trained in neonatal and pediatric resuscitation according to European Resuscitation Council (ERC) guidelines [5,6]. The participating paramedics had all completed at least three years of medical school education at the time of participation, and had received advanced emergency medical technician training [15], as well as at least three weeks of pediatric airway management training during a clinical elective at the Department of Pediatric Anesthesia [16]. We included all participants with informed consent into the study, and an absence thereof was the theoretical exclusion criterion.

The present study was designed as a pilot study, because no data on sensitivity and specificity were available. Therefore, no sample size calculation was performed. We hypothesized that a sample size of 40 would be representative for the present study [17].

### 2.2. Study Protocol

The participants acted as their own control and were asked to perform adequate ventilation for 90 s each. By throwing dice, the participants were randomized to either use two or five fingers first. For the two-finger technique, participants were instructed to use their thumb and index finger. For the five-finger technique, the SIB had to be compressed with the whole hand. Between both of the evaluation sessions, the participants were allowed to rest for five minutes so as to reduce the potential bias due to fatigue. No verbal feedback was given to the participants.

### 2.3. Manikin

A Laerdal Resusci Baby manikin (Laerdal Medical, Stavanger, Norway) was modified by removing the lung and stomach bags, and positioning a 0.5 L test lung (Dräger, Lubeck, Germany) into the chest, so that the chest excursions were similar to a standard training manikin. The test lung was connected to the mouth with non-distensible tubing and an airtight seal. When pressurized to 30 cm H_2_O, the maximal lung volume in our infant model was approximately 90 mL.

### 2.4. Ventilation Device

**Figure 1:**
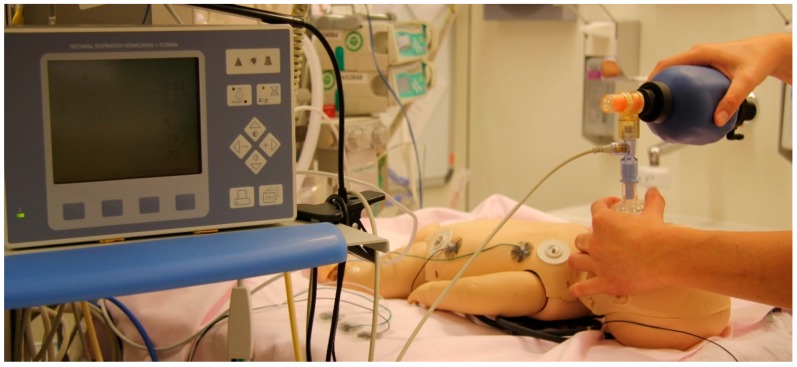
The experimental set-up consisted of a modified leak-free baby manikin, a self-inflating bag, and a respiratory function monitor.

The Ambu Baby R Resuscitator (Ambu, Ballerup, Denmark), a SIB with a maximal tidal volume of approximately 300 mL, was used. A silicon mask size 0/1 (Laerdal Medical, Stavanger, Norway) was connected to the SIB. The complete set-up is presented in Figure 1.

### 2.5. Respiratory Function Monitor (RFM)

A Florian Neonatal RFM (Acutronic Medical Systems, Zug, Switzerland) was used to measure the ventilation parameters. A hot-wire anemometer flow sensor was placed between the mask and SIB. The V_T_ was calculated by integrating the flow signals. The mask leak was expressed as a percentage of the inspired V_T_. During the recordings, participants were blinded to the screen of the RFM.

### 2.6. Data Acquisition and Analysis

The gas flow, V_T_, and airway pressure data were recorded at 200 Hz using a computer with α-Trace Digital MM (Best Medical Systems, Vienna, Austria) multi-channel system. We collected the gender, hand size (palmar distance from os pisiforme to distal end of second index finger), and shoe size (Continental European system) of the participants anonymously. Shoe size was evaluated in order to prove its potential use as a surrogate parameter for hand size.

### 2.7. Statistical Analysis

We primarily aimed to investigate the effect of a two- vs. five-finger BVM ventilation techniques on effective tidal volume (V_Teff_). To account for any possible problems at the beginning of each ventilation session, only the last 60 s of the recorded 90 s were analysed. We first calculated each respiratory cycle and then the mean for each participant for the following parameters: the delivered tidal volume (V_Tdel_) (mL), total inspiratory volume at the BVM, V_Teff_ (mL), total expiratory volume at the BVM, and mask leak (%), as $\frac{V_{\mathrm{Tdel}}-T_{\mathrm{Teff}}}{V_{\mathrm{Tdel}}}$. Furthermore, we calculated the ventilation rate (min^−1^) and ventilatory minute volume (L/min) as follows: $V_{\mathrm{Teff}}*\mathrm{ventilation}\;\mathrm{rate}$.

We expressed values as the mean ± standard deviation, median (interquartile range), or count (proportion), wherever appropriate. Firstly, we performed the Spearman correlation and non-parametric testing for the possible bivariate association between the assessed parameters. Then, we analysed the influence of the baseline parameters (gender, hand size, shoe size, and pediatric intensive care nurses vs. paramedics) on V_Teff_ using the two-finger technique vs. the five-finger technique by general linear modelling.

To guide future trials, we performed a post hoc sample size calculation using G*Power (Heinrich Heine University, Düsseldorf, Germany) to calculate the number of individuals needed for the measurement of a relevant difference in V_Tdel_, which was considered as a difference of at least 15% between both methods. A two-sided *p*-value of <0.05 was considered significant. We used IBM^®^ SPSS 20 (Armonk, NY, USA) for the data analysis.

The whole study was conducted from September to October 2014 at the Medical University of Graz, Graz, Austria. We did not change the protocol or the definition of the outcome measures during or after the conduction of the study.

## 3. Results

**Figure 2:**
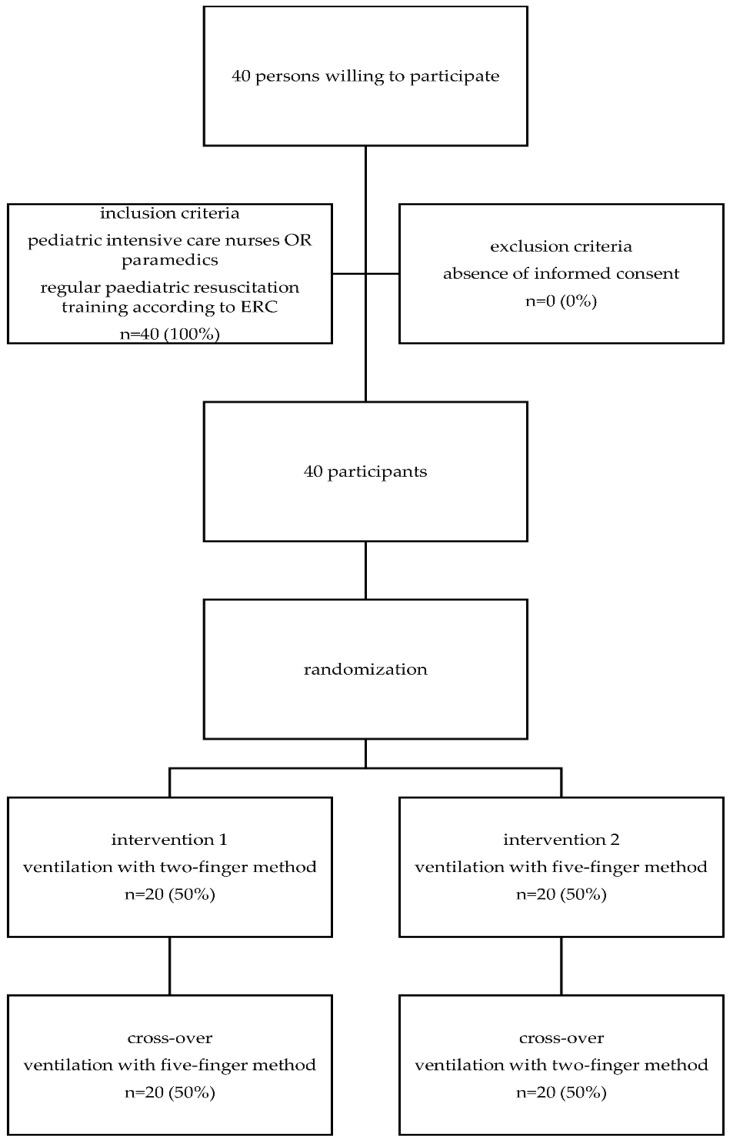
Study flow diagram. ERC—European Resuscitation Council.

A flow diagram can be found in Figure 2. There were 20 pediatric intensive care nurses and 20 paramedics that participated in the study. Of the participants, 25 (62.5%) were female, with an overall mean shoe size of 40.0 ± 2.7 Paris points and a median hand size of 17.5 (17–18.5) cm. A total of 5401 inflations were analysed, including 2796 inflations with two fingers and 2605 inflations with five fingers. All of the participants completed the entire protocol.

### 3.1. Effective and Delivered Tidal Volume (V_Teff_ and V_Tdel_)

**Figure 3:**
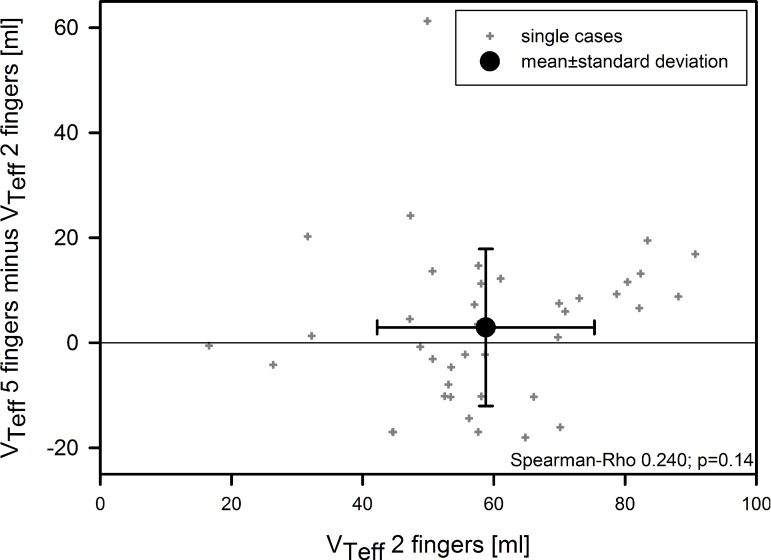
Comparison of effective tidal volume (V_Teff_) between two-finger ventilation and five-finger ventilation.

The V_Tdel_ was significantly lower during the two-finger technique compared with the five-finger technique (87.7 ± 15.5 vs. 96.1 ± 19.4 mL, respectively; *p* < 0.01; as seen in Table 1). The mask leak was also significantly reduced for the two-finger technique (27.9 ± 22.4 vs. 34.4 ± 24.5 mL; *p* = 0.02). As a result, the V_Teff_ did not differ significantly between the two-finger and five-finger techniques (58.8 ± 16.6 vs. 61.7 ± 23.9 mL, respectively; *p* = 0.35; Table 1). V_Teff_ was similar in the pediatric intensive care nurses and paramedics. The difference between two- and five-finger techniques tended to be more pronounced at higher tidal volumes, but this correlation was not significant (Spearman’s ρ 0.24; *p* = 0.14; Figure 3).

### 3.2. Ventilation Rate and Ventilatory Minute Volume

We observed a statistically significant difference with higher ventilation rates for the two-finger technique compared with the five-finger technique (46.6 ± 16.9 vs. 43.4 ± 16.4 min^−1^, respectively; *p* < 0.01). The minute ventilation was similar in both groups (2.76 ± 1.24 vs. 2.71 ± 1.59 L/min, respectively; absolute difference 0.05 ± 0.71 L/min, respectively; *p* = 0.40).

### 3.3. Influence of Anthropometric Data on V_Teff_

The correlations of the hand and shoe sizes with V_Teff_ were low and non-significant (ρ < 0.1 and *p* > 0.1 for both).

### 3.4. Multivariate Analysis for V_Teff_

We did not find any significant interactions between the baseline parameters (gender, hand size, shoe size, and pediatric intensive care nurses vs. paramedics) and our primary endpoint (V_Teff_).

### 3.5. Post-Hoc Sample Size Calculation

As the mean V_Teff_ in the two-finger technique was 58.8 mL, the mean V_Teff_ would have to be at least 67.6 mL in the five-finger technique in order to be clinically relevant. We performed a post hoc sample-size calculation with a normalized effect size of 0.481 (considering a standard deviation of 20 mL). To achieve a power of 80%, a sample size of at least 36 participants would be necessary to detect a relevant difference between both methods.

## 4. Discussion

To the best of our knowledge, this is the first study comparing the number of fingers used during mask ventilation using a SIB, and their effect on V_T_ delivery. We did not observe any differences in V_Teff_ between the two-finger and five-finger technique during mask ventilation. Our results support the current ERC guidelines, which do not offer any specific recommendations regarding the number of fingers used for mask ventilation [5,6,18]. The ERC guidelines only state that insufflations should result in a chest rise so as to ensure adequate V_T_ delivery. Other recommendations, such as the aforementioned “rule of thumb”, lack any scientific evidence and should therefore not be used. Healthcare personnel in training may therefore be taught to use as many fingers as they want to on their SIB, as long as the clinical features signalize adequate ventilation.

Although the five-finger technique may not necessarily lead to a higher V_Teff_, the mask leak and V_Tdel_ were shown to be higher compared with the two-finger technique. We assume that the ventilation pressures during the BVM ventilation are higher using the five-finger technique, and therefore result in a higher mask leak. By recognizing an adequate chest rise early on, healthcare professionals may interrupt the inflation immediately, and thus avoid an overinflation of the lungs, even when delivering higher inspiration pressures.

We found statistically significant differences in the ventilation rates between the two-finger and five-finger techniques. This finding may be of questionable clinical relevance because of the small absolute difference, especially when considering that the values were within the normal range of the current guideline recommendations in both groups.

Besides analysing the effect of the number of fingers used, we studied the effect of anthropometric measures and gender on tidal volumes. In certain resuscitation situations during our clinical careers, we had the impression that men with large hands (and feet) would deliver higher tidal volumes during neonatal or infant resuscitation. However, this hypothesis needs to be rejected because of a negligible correlation between hand/shoe size and V_Teff_ in our study.

### Limitations

Although we tried to design our simulation model to be as realistic as possible, and the study was large enough to detect clinically relevant differences, the results may not fully reflect a real clinical setting. As a result of the modifications to our resuscitation manikin in order to ensure air tightness, the absolute V_Teff_ in our model was significantly higher and cannot be compared to the tidal volumes in actual neonates or infants. However, we could determine changes in the tidal volumes between both of the ventilation techniques easily, because each volunteer served as his/her own control.

Furthermore, in this study, we only assessed BVM ventilation. Therefore, our results may not be transferable to invasive ventilation or non-invasive ventilation using a T-piece or another ventilatory device.

Thirdly, we analyzed BVM ventilation in the participants that received regular training in neonatal and/or pediatric resuscitation. Therefore, this study is not generalizable to other personnel who are trained less often.

Lastly, we aimed to minimize dead space, and therefore did not assess the differences in pressure between both techniques, which may also have had major implications on the clinical outcome.

## 5. Conclusions

This study shows that, in an infant model, the use of two or five fingers for BVM ventilation leads to a similar V_T_ delivery, regardless of the hand or shoe size of the performing personnel.

## Figures and Tables

**Table 1:** Comparison of tidal volumes, leak, and ventilation rate between two-finger vs. five-finger techniques.

	Two-Finger Technique	Five-Finger Technique	Absolute Difference	*p*-Value
V_Teff_ (mL)	58.8 ± 16.6	61.7 ± 23.9	+2.9 ± 15.9	0.35
V_Tdel_ (mL)	87.7 ± 15.5	96.1 ± 19.4	+9.4 ± 12.7	<0.01 *
Mask leak (mL)	27.9 ± 22.4	34.4 ± 24.5	+6.5 ± 16.7	0.02 *
Mask leak (%)	30.0 ± 21.0	34.6 ± 23.0	+4.5 ± 16.0	0.02 *
Ventilation rate (min^−1^)	46.6 ± 16.9	43.4 ± 16.4	−3.2 ± 6.1	<0.01 *
Ventilatory minute volume (L/min)	2.76 ± 1.24	2.71 ± 1.59	−0.05 ± 0.71	0.40

V_Teff_—effective tidal volume; V_Tdel_—delivered tidal volume; * *p* < 0.05.
